# Can China’s national fitness policy contribute to achieving universal health? Analysis based on the three-dimensional framework

**DOI:** 10.3389/fpubh.2025.1610070

**Published:** 2025-08-18

**Authors:** Defeng Dong, Bing He, Jianda Kong, Aitong Zhou, Mei Lv, Dianbo Zhang, Chen Dong

**Affiliations:** ^1^School of Sport Management, Shandong Sport University, Jinan, China; ^2^School of Business, Jiangsu Ocean University, Lianyungang, China; ^3^College of Sports Science, Qufu Normal University, Qufu, China; ^4^School of Management, Beijing Sport University, Beijing, China; ^5^Physical Education Department, Shandong Women’s University, Jinan, China

**Keywords:** national fitness, health for all, health promotion, three-dimensional analytical framework, policy text analysis

## Abstract

**Introduction:**

A lack of physical activity is widely regarded as an important factor contributing to the increase in non-communicable diseases and mortality rates. To improve the physical fitness and health levels of its citizens, the Chinese government has launched the National Fitness Program (NFP) to promote public health and well-being. However, the mechanisms and performance of these policies in promoting public health still require further exploration.

**Methods:**

This paper conducts a systematic analysis and evaluation of the NFP from 1995 to 2025 from three dimensions: policy themes, policy tools, and policy consistency, using text analysis, content analysis, and the Policy Modeling Consistency (PMC) model.

**Results and conclusion:**

The results show that the core themes of the NFP focus on infrastructure development, mass sports activities, and the public health service system. In terms of policy tools, there is an overall structural imbalance and internal disarray, with environmental policy tools dominating, supplemented by supply-side policies, while demand-side policies are insufficient. The level of policy consistency is good, and the design is relatively scientific and reasonable. However, there are significant differences between policies, and some indicators need improvement. Therefore, we recommend: enhancing cross-departmental, cross-disciplinary, and cross-regional cooperation, emphasizing evidence-based decision-making; promoting the balanced development of national fitness, increasing the inclusivity of policies, and focusing on the rights of special groups; Increasing incentive measures to encourage social participation; increasing the use of demand-oriented policy tools; improving the policy evaluation system to achieve dynamic optimization of policies.

## 1 Introduction

Health refers to a state of complete physical, mental, and social well-being (1, 2), and is the most fundamental need and right of every individual (3). It is also the prerequisite for achieving sustainable social development (4, 5). Physical activity (PA), as a key pathway to improving public health levels (6), addressing population aging, alleviating overweight and obesity, regulating mental health, preventing non-communicable diseases, and promoting social sustainability (7), has attracted increasing attention from countries and international organizations (8, 9). It has also become one of the most active and effective ways to achieve Sustainable Development Goal 3 (Good Health and Well-being) (2, 10, 11). The latest data released by the World Health Organization (WHO) in 2024 indicates that 31% of adults and 80% of adolescents globally fail to meet the recommended physical activity levels (12, 13), highlighting the worsening global trend of insufficient physical activity. If this trend continues, the proportion of the global population failing to meet the standards is expected to rise to 35% by 2030, far exceeding the 15% reduction target set by the Global Physical Activity Action Plan (2018–2030). And most countries are off track to meet the Sustainable Development Goals (SDG) (13). A large body of research indicates that insufficient PA is a decisive factor in the onset of non-communicable diseases (14), The further increase in the population with insufficient PA not only poses higher demands on the systemic responsiveness of national health promotion policies, but also will lead to a greater global health burden (15).

The Adelaide Recommendations on Healthy Public Policy state that healthy public policies can create a supportive environment for health promotion actions (16). A large body of research also highlights the necessity and impact of effective PA policies (17, 18). Therefore, in response to global health risks, governments worldwide have continuously strengthened the institutional integration and governance capacity of their policy systems, gradually building a policy framework that promotes the collaboration of “exercise—health—social welfare” (19). In practice, several countries have successively introduced multidimensional intervention systems that include action recommendations, resource allocation, and institutional guarantees. Taking the United States as an example, its Physical Activity Guidelines for Americans have evolved into a national-level behavioral guideline covering the entire life cycle, with population-based recommendations, and have integrated physical activity intervention systems into primary health services and chronic disease prevention through federal public health programs (20). Canada’s 24-Hour Movement Guidelines integrate physical activity, sedentary behavior, and sleep behavior, promoting individual health management within a multi-behavior governance framework that extends into daily life scenarios, reflecting a paradigm shift from single-behavior advocacy to composite behavior governance (21). At the institutional level, Nordic countries commonly adopt a policy structure of “multi-departmental coordination—local co-governance—citizen participation” (22), emphasizing collaborative governance across education, urban construction, transportation, and health sectors, and building cross-sector policy coordination platforms and performance accountability mechanisms (19). Japan is the first Asian country to establish guidelines for physical activities, promoting health and addressing the challenges of population aging. The Japanese government has issued a series of policies and regulations, such as the enactment of the Basic Act on Sport and the Sports Revitalization Plan, to ensure the smooth implementation of physical activity programs (23). Japan’s Health Japan 21 national strategy, in response to population aging, is based on a three-level intervention pathway of “community—family—individual,” creating a comprehensive policy network covering medical prevention, social support, and health promotion (24). The above policy pathways indicate that the international sports health policy system is undergoing an evolutionary shift from “exercise advocacy-based” to “structural integration-based” and from “individual directive-based” to “environmental construction-based.” Its core features include a diversified combination of policy tools, a networked governance structure, and a systematic evaluation mechanism, reflecting the growing development of health governance toward equity, evidence-based approaches, and integrated governance (25).

As the largest developing country, China faces the global trend of shifting the health risk spectrum from acute infectious diseases to non-communicable diseases, which brings multiple structural pressures on its public health system. In 2021, 10.64 million people in China died from chronic diseases, accounting for 91% of the total deaths. A recent study published in The Lancet shows that China has become the country with the largest population of overweight and obese individuals (402 million), far exceeding India, which ranks second (180 million) (26). These data reflect the severe challenges China currently faces, including the high incidence of chronic diseases and the promotion of healthy lifestyles. Meanwhile, with the growing demand for fitness among residents, the insufficiency and imbalance in the supply of public fitness services have become urgent issues that need attention (27). Against the backdrop of escalating health risks and increasingly prominent structural contradictions, the Chinese government launched the National Fitness Program (NFP) in 1995 to improve the health level of all citizens. Since then, National Fitness is no longer solely a specialized matter within the sports system, but has become a strategic institutional arrangement aimed at addressing major public health risks, improving public health literacy, and alleviating the burden on the basic healthcare system. From the perspective of policy drivers, this shift reflects the gradual reorganization of national governance objectives from a “disease-centered” focus to a “health-centered” approach. It also marks the integration of sports policy into the core domain of the national public health governance structure, serving multiple goals such as disease prevention, health promotion, and equitable governance. The Chinese government has continuously strengthened the strategic responsiveness of national health policies, building a public health governance system centered around the NFP. Since the release of the National Fitness Program Outline in 1995, several important policy documents have been issued, such as the National Fitness Program (2011–2015) and the National Fitness Program (2021–2025). Guided by the Healthy China 2030 Plan, the fitness strategy has been deeply integrated with basic public health, medical security, and aging response policies. This demonstrates strong top-level coordination and goal-oriented capacity (28).

In recent years, with the continued advancement of the NFP, domestic research has made significant progress in policy system analysis and historical evolution. Professor Tien-Chin Tan, through qualitative analysis, found that under China’s top-down policy system, disparities in economic and cultural development between regions have caused a disconnect between central policy goals and local implementation, leading to uneven regional development (29). Zheng Liu points out that although cross-disciplinary integration has brought new opportunities to the development of public fitness in China, the gap in the level of mass sports development between urban and rural areas, as well as between regions, remains a major challenge to the equalization of public fitness services (30). In terms of policy text analysis, Mingxing Yu systematically analyzed and evaluated 13 national fitness policies from 1995 to 2021 by constructing a two-dimensional analytical framework of policy objectives and policy tools, revealing issues such as the insufficient attention given by government departments to the construction of content systems (31). Existing studies primarily rely on theoretical frameworks such as policy process theory and historical institutionalism, focusing on the evolutionary pathways of policies and the selection of policy tools, providing critical perspectives for understanding policy evolution and governance logic (32, 33). However, most studies tend to focus on qualitative analysis and lack significant progress in quantitative modeling. There is no existing framework that combines qualitative analysis with a systematic approach based on large-scale policy texts, making it difficult to reveal the inherent coupling relationships between potential policy themes, policy tool configurations, and design logic (34). For example, some scholars have attempted to quantitatively assess the health effects of the NFP from an outcome perspective, verifying its positive impact on public health levels. However, such studies focus on “policy outputs” and lack in-depth exploration of the structural consistency, logical rigor, and implementation mechanisms of policy design itself, leaving the core question of “why it is effective or not” unresolved (35). Foreign scholars generally emphasize the systematic integration of sports policies with health strategies in health promotion research, highlighting that sports policies should be embedded within the institutional logic of national health governance systems (36). Some scholars have used the Health-Enhancing Physical Activity Policy Analysis Tool developed by the WHO to analyze and evaluate health promotion policies (37). Research on foreign physical activity policies covers marginalized populations such as adolescents (38), women (39), the older adult (40), and people with disabilities (41), who are often overlooked. The research methods place greater emphasis on interdisciplinary integration and the use of mixed-methods approaches (42, 43) These studies provide valuable references for promoting equitable, systemically integrated health policies and offer theoretical support for the structural analysis perspective developed in this study.

In the related research on policy system analysis, scholars have begun to construct two-dimensional and three-dimensional policy analysis frameworks, using a combination of methods for multi-angle mixed analysis of policy texts. For example, Xiaokun Sun constructed a three-dimensional analysis framework of “policy goals - policy tools - value chain” to analyze 126 Chinese smart aging policies (44). Weiwei Chang and Qi Meng both constructed a “goals-tools-effectiveness” three-dimensional framework to perform quantitative analysis on China’s higher education policies and digital sports bridging policies, respectively (45, 46). This paper, based on existing policy analysis frameworks proposed in the literature, introduces the policy theme dimension and constructs a three-dimensional analytical framework of “policy themes - policy tools - policy consistency” to conduct a multi-dimensional systematic analysis of China’s NFP. Although this framework has some surface similarities with the classic “structure-process-outcome” model, its theoretical logic integrates the “Health in All Policies” approach with multi-sector governance theory, emphasizing the importance of theme integration, tool diversification, and cross-departmental structural collaboration.

**Figure 1:**
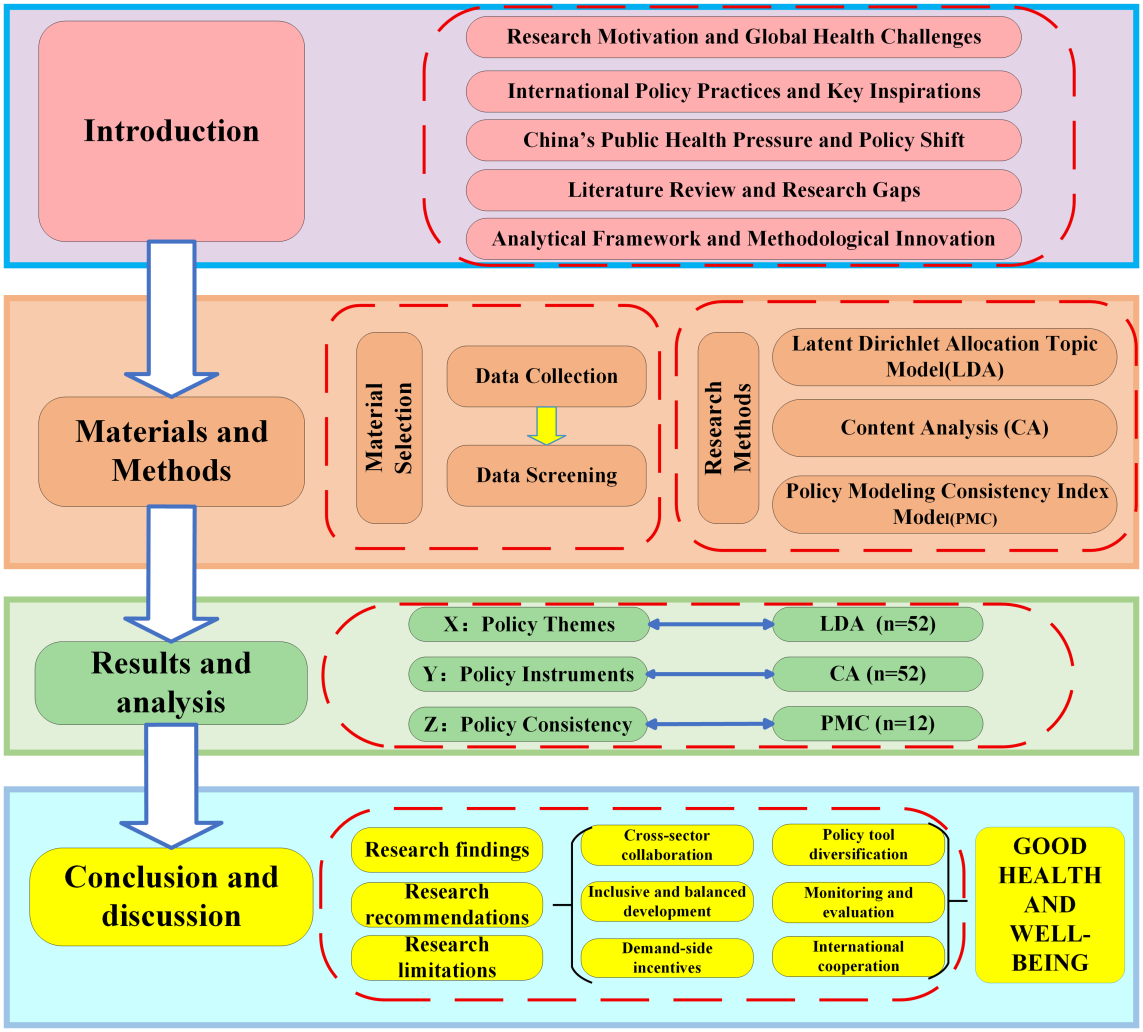
Research design. Adapted with permission from Xu and Hu (73).

Unlike previous studies that evaluate the National Fitness Policy (NFP), this study systematically reveals the structural characteristics and operational logic of China’s NFP through cross-validation using multiple methods and collaborative analysis with multiple models. In terms of research methodology, this study uses the Latent Dirichlet Allocation (LDA) topic model to identify core issues and focal points within the policy texts released at the national level from 1995 to 2025, mapping the policy direction from a content perspective. Additionally, NVivo 15 software is used to code and classify policy tools within the policy texts, exploring their tool configuration patterns. Finally, the Policy Modeling Consistency (PMC) model is established to assess the structural consistency of typical policies, measuring the logical integrity and systemic coupling of policies at the macro level (see Figure 1). The innovation of this study lies in breaking through the traditional fragmented, descriptive, and static approach in NFP research, attempting to transform policy texts into identifiable and modellable structural objects by combining qualitative and quantitative research, thus advancing the analysis of NFP toward a systematic “structure-mechanism-performance” trinity framework. In terms of research methods, this study leverages the complementary advantages of text analysis, content analysis, and the PMC index model, helping to uncover the evolving patterns and content structures within policy texts. At the same time, it provides theoretical support and empirical evidence for the collaborative governance, scientific evaluation, and optimization of the NFP system. This work addresses the functional role of the NFP within national health strategies, promoting health equity, resource optimization, and scientific governance, while contributing to the high-quality development of the Healthy China strategy. The findings are of significant theoretical and practical importance for policymakers, administrators, stakeholders, and researchers, and aim to provide policy design references for countries facing insufficient physical activity among their populations.

## 2 Materials and methods

### 2.1 Data sources and collection

#### 2.1.1 Data sources

The NFP is a normative institutional arrangement formulated by political parties and state agencies to meet the physical activity needs of all citizens. Its essence lies in the allocation of public sports resources and governance mechanisms by the state (32). To comprehensively and systematically reveal the structural evolution and design logic of the NFP, this study sets the sample period from January 1, 1995, to January 1, 2025, covering the major policy development stages from the release of the National Fitness Program Outline (1995) to the present. Policy texts were initially collected by searching for terms such as “NFP,” “Mass Sports” and “Public Sports Services” on the official websites of the State Council, the General Administration of Sport of China, and the Ministry of Education, with additional searches conducted using the Peking University Law Database (China’s legal information database). After de-duplication and cleaning, a total of 4,017 policy documents were obtained, with 544 from the national level and 3,473 from the local level. The types of texts involved include formal announcements, laws and regulations, planning outlines, implementation opinions, notices, work points, departmental plans, and other categories, covering various forms of publication within the NFP system. To ensure the comprehensiveness and authority of the data, the collection and screening of policy texts were carried out by three researchers with a background in policy studies, followed by multiple rounds of cross-checking to identify and correct any omissions.

#### 2.1.2 Data screening

To ensure the scientific validity, relevance, and authority of the sample, this study systematically screened the 4,017 policy documents obtained, following the three principles outlined below: (1) Comprehensiveness Principle: Prioritize the inclusion of formal policy texts with complete content, standardized structure, and strategic or institutional functions, including laws, regulations, development plans, implementation opinions, special programs, and departmental notices, while excluding non-representative operational or procedural documents such as meeting notices, summary materials, and training documents. (2) Relevance Principle: Based on the content of the texts, only those focusing explicitly on “National Fitness” were retained, while excluding documents that mention NFP as a supplementary background or peripheral statement. (3) Authority Principle: Considering the highly centralized nature of China’s policy system, where local policies are often derived from the re-deployment and adaptation of central policies (35), this study focuses on national-level policy documents issued by the Central Committee of the Communist Party of China, the State Council, and the ministries of the State Council, ensuring that the research sample maintains high consistency in terms of publishing authority, policy status, and structural stability (47, 48). Based on this, after two rounds of screening and manual cross-verification, 52 national-level policy documents were retained as the research sample (see Table 1). These 52 texts include the 1995 National Fitness Program Outline, various Five-Year Plans (such as 2011–2015 and 2021–2025), and key strategic or normative documents from each phase, covering core policy categories such as laws, plans, implementation opinions, work points, and special programs, constituting the main components of the NFP system. No influential central policy documents were found to be omitted during the screening process.

**Table 1:** Overview of the national fitness policy texts (partial).

Number	Title	Time	Publishing department
1	Outline of the National Fitness Program	June,1995	State Council
2	Opinions of the State Physical Culture and Sports Commission on Implementing the “1–2-1 Project” for the Outline of the National Fitness Program	June,1995	State Physical Cultural Administration
3	Opinions on Implementing the Outline of the National Fitness Program	July,1995	Ministry of Education
…	…	…	…
50	Opinions on Promoting National Fitness, Sports Consumption, and High-Quality Development of the Sports Industry	September,2019	State Council
51	National Fitness Program (2021–2025)	July,2021	State Council
52	Key Points of Work on Mass Sports in 2024	February,2024	State Physical Cultural Administration

### 2.2 Research procedure and design

Based on the content, structural characteristics, and basic attributes of the NFP texts, a three-dimensional analytical framework for China’s NFP is constructed from three dimensions: policy themes (X dimension), policy tools (Y dimension), and policy consistency (Z dimension; see Figure 1) (73). Specifically, the X dimension uses the LDA topic model to visually present the potential themes and future development trends of the NFP, providing a basis for the establishment of secondary tools in the policy tools dimension and for defining the evaluation criteria of the PMC model. The Y dimension employs policy tool theory and uses NVivo 15 software to qualitatively analyze the use of policy tools. Whether the tools are used in a balanced manner will impact the effectiveness of the policy. The Z dimension uses the PMC model to construct an evaluation index system for the NFP, assessing the consistency of the policy and its logical alignment with the policy themes, thus enabling a quantitative and objective evaluation of the policy texts. Therefore, this study combines qualitative and quantitative methods to analyze and evaluate China’s NFP from multiple dimensions, effectively neutralizing the subjectivity of qualitative research and the objectivity of quantitative research, making the research findings more scientific.

#### 2.2.1 Policy themes (X dimension)

Policy themes directly reflect the goal orientation of the policy, and analyzing policy themes helps better understand the deeper connotations of the policy. This study uses the LDA topic model to perform topic modeling analysis of the NFP. The LDA topic model, proposed by Blei, is a text mining tool based on a three-layer Bayesian probabilistic framework (documents, topics, and words) (49). The principle is to train the model using unsupervised machine learning, which identifies the latent themes of the policy from a large volume of policy texts (50), effectively reducing the randomness and complexity of manual coding (51).

#### 2.2.2 Policy tools dimension (Y dimension)

Policy tools are methods and means adopted by policy actors to solve practical problems, implement policy plans, and achieve policy goals. They represent the pathways and mechanisms that translate the government’s substantive goals into concrete actions, reflecting the will of the policy actors (52). By analyzing the use of policy tools, this not only provides scientific evidence for policy design for policy makers, but also offers a rational perspective for policy stakeholders and policy researchers to understand the policy. Policy tool theory dates back to the mid-20th century, with scholars such as Lowi proposing various theoretical frameworks based on different policy execution characteristics. As research developed, this field gradually formed a more systematic body of knowledge. For example, Howlett categorized policy tools into three types: coercive, hybrid, and voluntary, based on the intensity of government intervention in the behavior of the target population (53). In this study, we referred to the classic tripartite classification proposed by Rothwell and Zegveld (54), dividing policy tools into supply-side, environmental, and demand-side categories. We also incorporated the research of domestic scholars, such as Juan Chang (55) and Xianliang Wang (56), to construct a policy tool classification system that covers the context of the NFP (see Table 2). Specifically, this study adopts a directed content analysis method, with theoretical support, to pre-define a complete coding framework that includes three main types of policy tools and their secondary subcategories (see Table 2). Subsequently, the research team used NVivo 15 software to systematically code 52 national-level NFP texts, identifying and counting the specific frequency and structural distribution of each policy tool within the texts, in order to reveal the characteristics and status of the tool configuration in the current NFP.

**Table 2:** Policy tool classification and explanations.

Tool type	Tool name	Connotation
Supply-based	Infrastructure	The construction and maintenance of necessary infrastructure for the Sports for All public service system.
	Talent Development	Enhancing talent cultivation and training systems to support the investment of human resources in public services.
	Technological Support	Promoting the use of technologies such as big data and artificial intelligence to support Sports for All programs.
	Financial Investment	The government provides financial support for the development of Sports for All through funding such as fiscal allocations and development funds.
	Physical-medical integration	Promoting the integration of sports and medical fields, fostering collaborative approaches that combine exercise and healthcare.
Environmental	Strategic Measures	Formulating feasible measures, methods, and strategies to promote the actual development of Sports for All.
	Goal Planning	The development goals and strategic plans for Sports for All.
	Regulatory Oversight	The formulation of laws, regulations, and supervision mechanisms to guide the orderly development of Sports for All.
	Tax Incentives	Government measures to improve or reduce taxes and fees for relevant institutions and enterprises.
	Financial Support	Relaxing loan and financing conditions, providing financial support for the sector.
Demand-based	Collaborative Development	Defining the roles of government departments and establishing collaborative mechanisms for joint governance.
	Outsourcing Services	The government entrusts social forces or third parties to provide fitness services or projects.
	Pilot Projects	Leading Sports for All development through the establishment of pilot projects for fitness programs.
	International Exchange	Engaging in exchange activities with other countries related to health promotion.
	Government Procurement	The government procures sports products or services from social enterprises or organizations.

#### 2.2.3 Policy consistency dimension (Z dimension)

Policy effectiveness refers to the actual impacts and effects demonstrated by policy implementation. It is a core indicator that reflects the effectiveness of the policy content and the execution, comprising two parts: content effectiveness and implementation effectiveness (45, 57). The degree of policy text consistency reflects, to some extent, the content effectiveness of the policy text, meaning that the higher the level of policy consistency, the higher the content effectiveness (57). However, it should be noted that the level of consistency does not directly reflect the effectiveness of policy implementation, as implementation effectiveness also involves feedback, monitoring, and adjustment mechanisms in actual operations, which goes beyond the scope of text-level evaluation. The evaluation of policy content effectiveness is an important component of public policy formulation and management. Common methods include Analytical Hierarchy Process (AHP), fuzzy comprehensive analysis, and entropy-weight method, which often carry strong subjectivity. To avoid the impact of subjective policy evaluations, this study constructs the PMC index model evaluation system based on the content of the policy texts, and performs a multi-dimensional, objective evaluation focused on policy consistency. The PMC index model, proposed by Ruiz Estrada based on the Omnia Mobilies hypothesis, emphasizes the widespread interconnections among all things. The innovation of this model lies in its broad consideration of variables, with no limits on the number of secondary variables and equal weights, which effectively reduces the impact of subjective biases and vagueness in policy evaluation, thereby enhancing the comprehensiveness and systematization of the evaluation (58).

### 2.3 Research methods

#### 2.3.1 Text analysis method

Text analysis is the process of extracting valuable information and latent patterns from unstructured textual data, aimed at parsing, classifying, extracting, and modeling text data. It is commonly used to reveal the thematic connections, structural logic, and evolution trends within policy documents. Among the various text analysis methods, topic modeling is a common approach to identifying the semantic features of literature. The goal of topic modeling is to automatically identify latent topic information from large amounts of unstructured text, and the LDA topic model is one of the most widely used methods in this area. The model was proposed by Blei et al. in 2003 and is an unsupervised probabilistic model based on a Bayesian framework (59). The LDA topic model assumes that documents are generated by a mixture of multiple latent topics, with each topic corresponding to a probability distribution over words. Its modeling process is akin to a ‘probabilistic generative game’: first, a random topic distribution is assigned to the document, then specific words are generated based on the topic’s word distribution. The model uses a multi-layer Dirichlet distribution (with prior constraints on the document-topic and topic-word distributions) to balance the sparsity and generalization of topics (60, 61). The advantage of this model lies in its ability to leverage unsupervised machine learning to extract latent topics from large-scale text data, helping researchers identify the core issues of the text as well as the evolution trends and interrelationships of the topics. Therefore, this study uses the LDA topic model to perform topic modeling analysis on 52 national-level NFP texts to identify the discourse structure and thematic evolution characteristics of the policy. The calculation formula of the LDA topic model is shown in Equation 1:


(1) P(d,w,z,θ,φ)=P(θ∣α)×∏P(z∣θ)×P(w∣z,φ)×∏P(φ∣β)

Equation 1 demonstrates the probabilistic relationships among documents, topics, and words. The word “*ω*” in document “d” is related to topic “z.” “θ” represents the topic distribution within the document, φ represents the distribution of words within a topic, and “*α*” and “*β*” are hyperparameters, usually with default values.

Following a thorough review of the policy texts, text preprocessing is performed as the initial step. The Jieba library is utilized for word segmentation of 52 policy documents. Subsequently, Python is employed to implement LDA modeling, using the Gibbs sampling method. The Coherence Model and LDA Model from the Gensim library are imported for this analysis (62). The hyperparameters “α” and “β” are set to 50/k and 0.1 respectively, and the number of iterations is 100. To ensure the reproducibility of the results, a fixed random seed (random_state = 42) was set for the model, and the consistency of the topic structure was validated through multiple runs of the model, enhancing the stability and reliability of the results.

**Figure 2:**
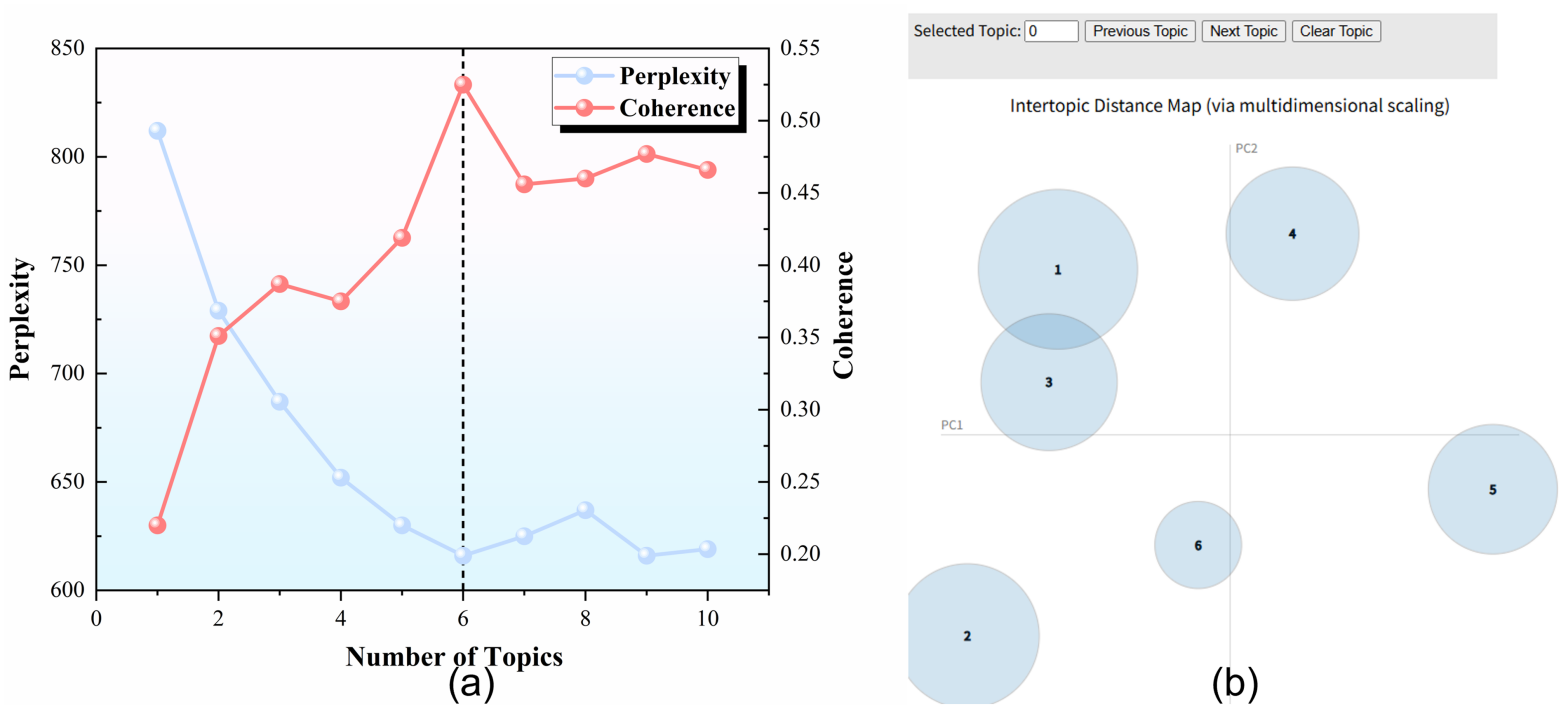
Evaluation and visualization of topic modeling results. **(a)** Perplexity and coherence scores for different numbers of topics. Lower perplexity and higher coherence indicate better model performance. As shown in the figure, the model performs optimally when the number of topics is 6 (indicated by the black dashed line). **(b)** Topic distance plot generated using the pyLDAvis tool. Each bubble represents a topic, and the size of the bubble indicates the proportion of the vocabulary for that topic. The distribution of topics is clear and non-overlapping, indicating good model discrimination with six topics.

In model evaluation, topic perplexity and coherence scores are two important indicators for assessing model quality. Topic perplexity measures the model’s predictive accuracy on the data; the lower the perplexity, the higher the accuracy of the latent topic model’s predictions. Coherence scores reflect the association and closeness between words; the higher the coherence score, the stronger the association between words, and the greater the interpretability of the model (63). As shown in Figure 2a, when the number of topics is 6, the model performs best, with higher coherence scores and lower perplexity. Visualization was also done using the PyLDAvis tool (see Figure 2b) (64). When the number of topics is 6, it was found that the six topics are well distributed and mutually independent in the two-dimensional space, further supporting the choice of six topics as the optimal model (65). Finally, the high-frequency keywords of each topic were identified through the “topic-word” probability distribution, and six categories of policy themes were summarized and refined.

#### 2.3.2 Content analysis method

Content analysis is a method that may be used with either qualitative or quantitative data and in an inductive or deductive way (66). This study adopts the content analysis method, using NVivo 15 software to code 52 national-level NFP texts. The analysis is based on a pre-defined policy tool classification system (see Table 2), which includes three main categories: supply-side, environmental, and demand-side tools, along with several specific subcategories. This method helps systematically extract the structural features and frequency distribution of tool usage from the policy texts while maintaining theoretical consistency.

To ensure consistency and reliability in coding, the research team organized unified training on software operation and variable determination criteria before formal coding. The coding work was independently completed by two researchers, and after the coding, the consistency was assessed using NVivo’s built-in Cohen’s Kappa coefficient tool. The results indicated that the Kappa coefficients for all primary coding nodes exceeded 0.81, meeting the high consistency standard, thereby demonstrating the strong reliability of the coding process in this study (67).

Additionally, the selected policy texts all originate from the official websites of the State Council, the General Administration of Sport of China, the Ministry of Education, and the Peking University Law Database, ensuring the authority and completeness of the text content. All samples are national-level policies, and after screening, they were included in the research scope, ensuring the representativeness of the data and the validity of the coding results.

#### 2.3.3 PMC index model

The PMC Index model was proposed by Ruiz Estrada, aiming to evaluate the design quality of policies by assessing the consistency of policy text content. The core goal of this model is to evaluate the consistency between various dimensions and variables within the policy text, thereby measuring the effectiveness and rationality of the policy design (58). The process of model construction and analytical evaluation includes: selecting and determining parameters for the two-level variables, establishing a multi-input–output table, calculating the PMC index, plotting the PMC surface, and policy evaluation, among other steps (68). The selection of variables in this model emphasizes “multi-perspective and comprehensive coverage,” which aligns with the objectives of this study (47). To ensure the scientific and effective selection of indicators, this study, based on the research of Estrada, Lizhen Shi (34), and other scholars, combined the modeling principles of the PMC index model, the results of text analysis, and expert interviews to establish 9 primary variables and 50 secondary variables (see Table 3).

**Table 3:** Policy effectiveness evaluation index system.

Primary variables	Secondary variables	Variable evaluation criteria
X1 policy nature	X1:1 Regulation	Does the policy reflect regulatory characteristics?
	X1:2 Advisory	Does the policy have advisory characteristics?
	X1:3 Guidance	Does the policy provide guidance?
	X1:4 Description	Does the policy describe the objectives?
	X1:5 Predictive	Does the policy have a predictive nature?
	X1:6 Diagnostic	Does the policy contain diagnostic elements?
X2 policy timeliness	X2:1 Long-Term	Does the policy involve long-term planning (≥5 years)?
	X2:2 Medium-Term	Does the policy involve medium-term planning (1–5 years)?
	X2:3 Short-Term	Does the policy involve short-term planning (within 1 year)?
X3 policy function	X3:1Coordination and Cooperation	Does the policy promote coordination and cooperation?
	X3:2 Regulatory Guidance	Does the policy have a regulatory guidance function?
	X3:3 Systemic Constraints	Does the policy contain institutional constraints?
	X3:4 Rights Protection	Does the policy provide rights protection?
	X3:5 Supervision and Monitoring	Does the policy include supervision and monitoring functions?
X4 policy focus	X4:1 Organizational Management	Does the policy address organizational management?
	X4:2 Competition and Training	Does the policy involve competition or training?
	X4:3 Cultural Promotion	Does the policy promote the culture of national fitness?
	X4:4 Facilities and Infrastructure	Does the policy address the development of sports facilities?
	X4:5 Fitness Guidance	Does the policy provide fitness guidance?
	X4:6 International Cooperation	Does the policy involve international exchanges and cooperation?
	X4:7 Integration of Sports and Medicine	Does the policy address the integration of sports and healthcare?
X5 policy evaluation	X5:1 Sufficient Evidence	Is the basis for the policy well-supported?
	X5:2 Clear Objectives	Are the policy’s objectives clear?
	X5:3 Scientific Plan	Is the policy plan scientifically designed?
	X5:4 Detailed Content	Does the policy provide detailed content?
	X5:5 Sustainable Development	Does the policy plan consider sustainability?
X6 policy audience	X6:1 Government	Is the government included as a policy audience?
	X6:2 Community	Is the community included as a policy audience?
	X6:3 Schools	Are schools included as a policy audience?
	X6:4 Enterprises	Are enterprises included as a policy audience?
	X6:5 Farmers	Are farmers included as a policy audience?
	X6:6 Women	Are women included as a policy audience?
	X6:7 Special Groups	Are special groups (e.g., disabled, older adult) included as a policy audience?
	X6:8 Sports Social Organizations	Are sports social organizations included as a policy audience?
X7 incentive measures	X7:1 Talent Incentives	Does the policy include talent incentive measures?
	X7:2 Tax Benefits	Does the policy offer tax benefits?
	X7:3 Financial Investment	Does the policy include financial investment?
	X7:4 Government Subsidies	Does the policy involve government subsidies?
	X7:5 Demonstration Projects	Does the policy include demonstration projects?
X8 issuing authority	X8:1 State Council	Is the policy issued by the State Council?
	X8:2 General Administration of Sport of China	Is the policy issued by the General Administration of Sport of China?
	X8:3 Other National Ministries	Is the policy issued by other national ministries?
	X8:4 Central Mass Organizations	Is the policy issued by central mass organizations?
X9 policy domains	X9:1 Economic	Does the policy involve the economic domain?
	X9:2 Social	Does the policy involve the social domain?
	X9:3 Political	Does the policy involve the political domain?
	X9:4 Cultural	Does the policy involve the cultural domain?
	X9:5 Educational	Does the policy involve the educational domain?
	X9:6 Technological	Does the policy involve the technological domain?
	X9:7 Environmental	Does the policy involve the environmental domain?

After establishing the evaluation system for policy effectiveness, calculate the PMC index values of each policy text. The specific calculation steps are as follows: ① Assign values to each secondary variable according to Equations 2, 3. Among them, the value range of each secondary variable is [0, 1]. Determine the value of the secondary indicator according to the content of the text. If the text contains the content of the secondary indicator, assign a value of 1, otherwise assign a value of 0; ② Calculate the value of each primary variable according to Equation 4, where t represents the primary variable, j represents the secondary variable, and T represents the number of secondary variables included in each primary variable; ③ Sum up the scores of each primary variable according to Equation 5, so as to calculate the PMC index of each national fitness policy.


(2) X∼N[0,1]

(3) X={XR:[0~1]}

(4) $$X_{t}(\sum_{j=1}^{n}\frac{X_{\mathrm{tj}}}{T(X_{\mathrm{tj}})}),t=1,2,3,\cdots \infty$$

(5) $$\begin{array}{l} \mathrm{PMC}=\\ \left[\begin{array}{c} X_{1}(\sum_{i=1}^{6}\frac{X_{1i}}{6})+X_{2}(\sum_{j=1}^{3}\frac{X_{2j}}{3})+X_{3}(\sum_{k=1}^{5}\frac{X_{3k}}{5})\\ +X_{4}(\sum_{l=1}^{7}\frac{X_{4l}}{7})+X_{5}(\sum_{m=1}^{5}\frac{X_{5m}}{5})+X_{6}(\sum_{n=1}^{8}\frac{X_{6n}}{8})+\\ X_{7}(\sum_{o=1}^{5}\frac{X_{7o}}{5})+X_{8}(\sum_{p=1}^{4}\frac{X_{8p}}{4})+X_{9}(\sum_{q}^{7}\frac{X_{9q}}{7}) \end{array}\right] \end{array}$$

According to the calculation results of the above PMC index, the PMC surface diagrams of each policy sample can be drawn. The PMC surface diagram intuitively shows the performance of the policy text in each dimension. Its degree of concavity and convexity reflects the internal consistency of the policy and the rationality of its structure. The protruding part of the surface indicates that the corresponding evaluation index of the policy has a higher score, and vice versa. The smaller the degree of concavity and convexity of the PMC surface diagram, it means that the internal consistency of the policy text is higher, the policy text is more comprehensive, and the policy is more effective (45). To draw the PMC surface diagram, it is first necessary to calculate the PMC matrix. According to the PMC index scores of each primary variable and Equation 6, a 3 × 3 PMC surface matrix is constructed, and the PMC surface diagram is drawn using Excel 2019 software. This article refers to Estrada’s policy classification standard to rate China’s national fitness policies (Table 4) (57).


(6) $$\mathrm{PMC}=[\begin{array}{ccc} X_{1} & X_{2} & X_{3}\\ X_{4} & X_{5} & X_{6}\\ X_{7} & X_{8} & X_{9} \end{array}]$$

**Table 4:** PMC index classification table.

PMC index	0–3.99	4–5.99	6–7.99	8–9
Level	Poor	Average	Good	perfect

**Figure 3:**
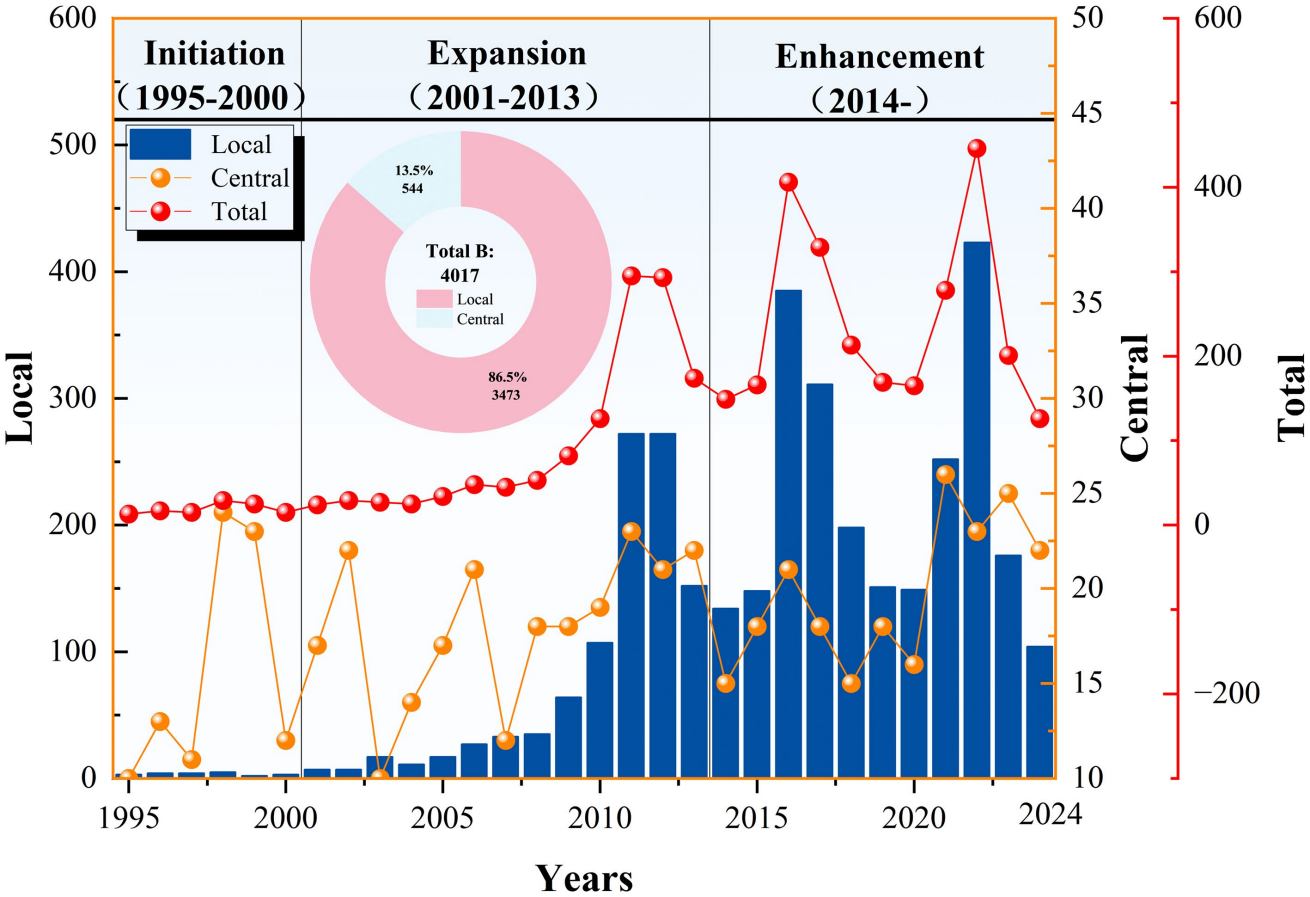
Number of national fitness policies released at different levels and stage distribution. This figure shows the number of NFPs released by central and local governments between 1995 and 2024, as well as their stage changes. The bar chart represents the number of local policies, the orange line indicates central policies, and the red line shows the total number. The background is divided into three stages: “Initiation Period,” “Expansion Period,” and “Improvement Period.” The pie chart reflects the cumulative policy composition, with 86.5% from local policies and 13.5% from central policies.

Due to the characteristics and operating mechanism of the PMC index model, which is not suitable for one-time evaluation of large sample sizes with low correlation, this study selects 12 representative policies from the 52 policy samples for evaluation, based on the principles of comparability, representativeness, and systematics. Specifically, this study selects four representative NFP texts from each of the three stages (see Figure 3), representing different development periods of the NFP: “Initiation Stage (1995–2000),” “Expansion Stage (2001–2015),” and “Improvement Stage (2016–2025)” (see Table 5). The selection criteria for these policies are based not only on their influence, historical significance, and feasibility but also on the policy’s periodicity and clarity of objectives, ensuring that the selected policies comprehensively reflect the characteristics and implementation effects of different developmental stages.

**Table 5:** Overview of representative national fitness policies.

	Number	Title	Time	Publishing department
Initiation	P1	Outline of the National Fitness Program	1995/6/20	State Council
	P2	Opinions of the State Physical Culture and Sports Commission on Implementing the “National Fitness 1–2-1 Project” in Accordance with the Outline of the National Fitness Program	1995/6/23	State Physical Cultural Administration
	P3	Opinions on Implementing the Outline of the National Fitness Program	1995/7/21	Ministry of Education
	P4	Outline of Sports Reform and Development (2001–2010)	2000/12/15	State Physical Cultural Administration
Expansion	P5	Plan for the Second - Phase Project (2001–2010) of the National Fitness Program Outline	2001/8/14	State Physical Cultural Administration
	P6	Plan for the Development of Mass Sports Undertakings during the 11th Five - Year Plan Period	2006/7/11	State Physical Cultural Administration
	P7	Regulations on National Fitness	2009/8/30	State Council
	P8	National Fitness Plan (2011–2015)	2011/2/15	State Council
Enhancement	P9	National Fitness Plan (2016–2020)	2016/6/15	State Physical Cultural Administration
	P10	Opinions on Promoting National Fitness and Sports Consumption to Boost High - Quality Development of the Sports Industry	2019/9/4	State Council
	P11	National Fitness Plan (2021–2025)	2021/7/18	State Council
	P12	Opinions on Constructing a Higher - Level Public Service System for National Fitness	2022/3/23	State Council

In summary, this study conducted a systematic analysis and evaluation of the NFP texts from multiple dimensions, combining various methods. The advantage of the research methods lies in the fact that the conclusions are based on actual data, incorporating standardized qualitative and quantitative analysis processes, which enhance the objectivity and logical coherence of the analysis. These methods are better suited to the complexity of policy text content and reduce the excessive subjective influence that may arise from pre-existing experiential viewpoints. The complementarity of the mixed research methods enhanced the scientific rigor of the study, and interdisciplinary research increased both the depth and breadth of the analysis. The intuitive visual analysis also made the research findings easier to understand and disseminate (47, 73).

## 3 Results and analysis

### 3.1 Policy evolution analysis

The number of policies reflects the formulation and release of the NFP, as well as the country’s awareness, emphasis, and progress in promoting national fitness (33). The number of NFP policies has shown a wave-like growth, with 544 national-level policy documents published, averaging 18.13 per year, and 3,473 local-level policies, averaging 115.77 per year (see Figure 3). The highest number of policies was released in 2022, with 23 national-level policies and 423 local-level policies, reflecting the Chinese government’ s emphasis on advancing NFP activities and improving public physical health. Based on the trend of policy release numbers and significant events, this study divides the evolution of China’ s NFP into three stages: (1) Initiation and Exploration Period (1995–2000): To promote mass sports activities and enhance the physical health of the public, the Chinese government issued the National Fitness Program Outline in 1995, marking the beginning of China’s “National Fitness Program.” (2) Full Promotion Period (2001–2013): Entering the 21st century, with improvements in the standard of living in China, to meet the demand for sports activities and address population aging, the National Fitness Program Outline Phase II (2001–2010) was issued, based on the first phase, marking the transition to the second phase, which focused on fully advancing the National Fitness Program. (3) Quality Enhancement and Efficiency Improvement Period (2014-present): The 2014 Opinions on Accelerating the Development of the Sports Industry and Promoting Sports Consumption proposed “actively advocating healthy living, promoting health in advance, and elevating the National Fitness Program to a national strategy.” Since then, the NFP has entered the stage of high-quality development. During this stage, fitness indicators steadily improved. The percentage of people who regularly engage in physical exercise increased from 33.9% in 2014 to 37.2% in 2021. The percentage of people meeting the National Physical Fitness Standards increased from 89.6% in 2014 to 90.4% in 2020. Health literacy for urban and rural residents increased from 8.80% in 2012 to 27.78% in 2022. Life expectancy increased from 75.5 years in 2012 to 78.2 years in 2021 (69).

### 3.2 Policy theme analysis (X dimension)

**Figure 4:**
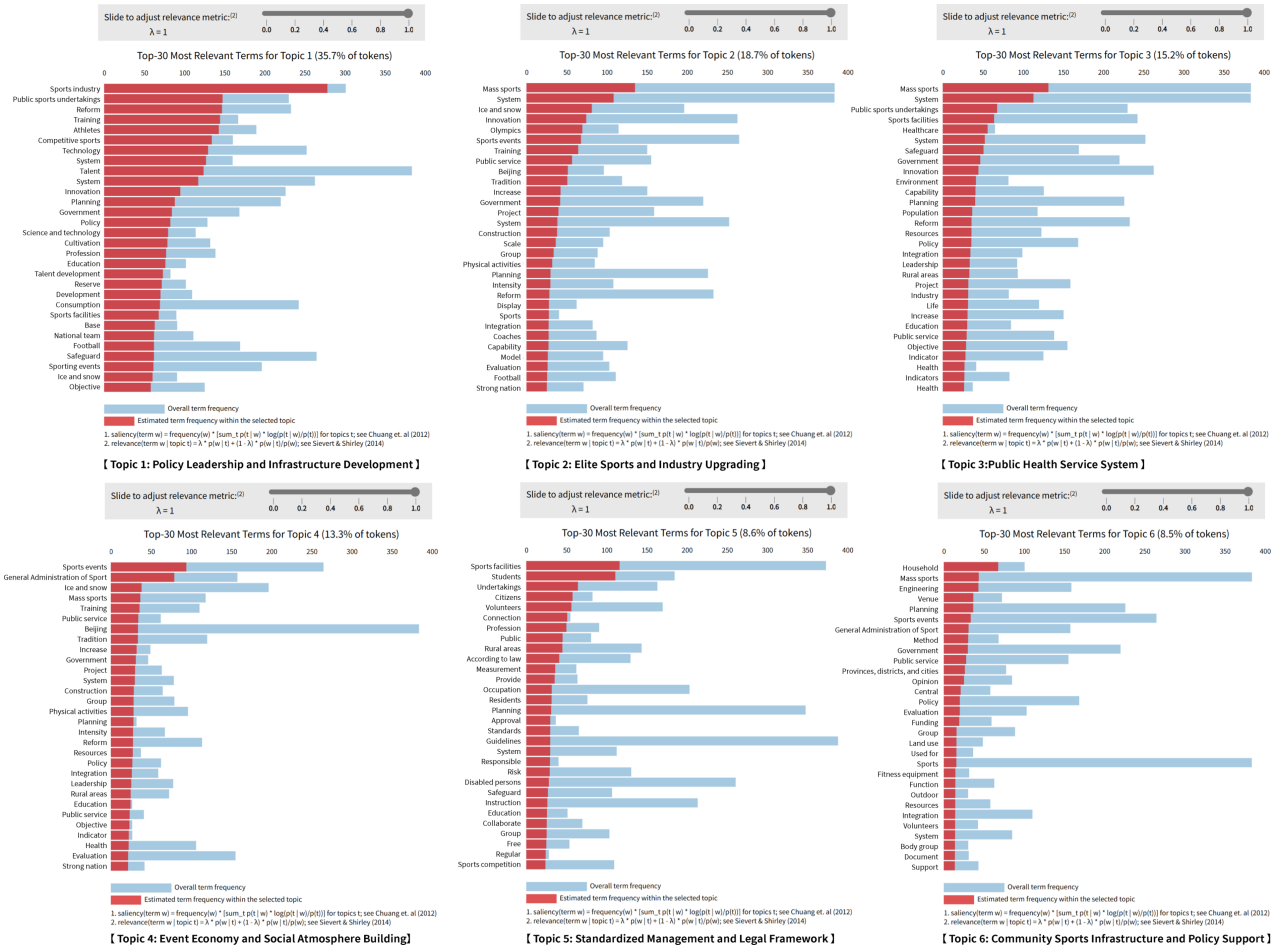
Distribution of high-frequency keywords for each theme.

To further reveal the semantic structure and evolutionary characteristics of the national-level NFP, this study applies the LDA topic model for topic modeling analysis on 52 policy texts released between 1995 and 2025. The analysis identified six main policy themes, covering various aspects of the NFP. The naming of each theme is based on the induction and summarization of its corresponding high-frequency keywords, with the frequency and correlation distribution of related terms shown in Figure 4. This helps to understand the semantic focus and logical connotations of the different policy themes.

**Figure 5:**
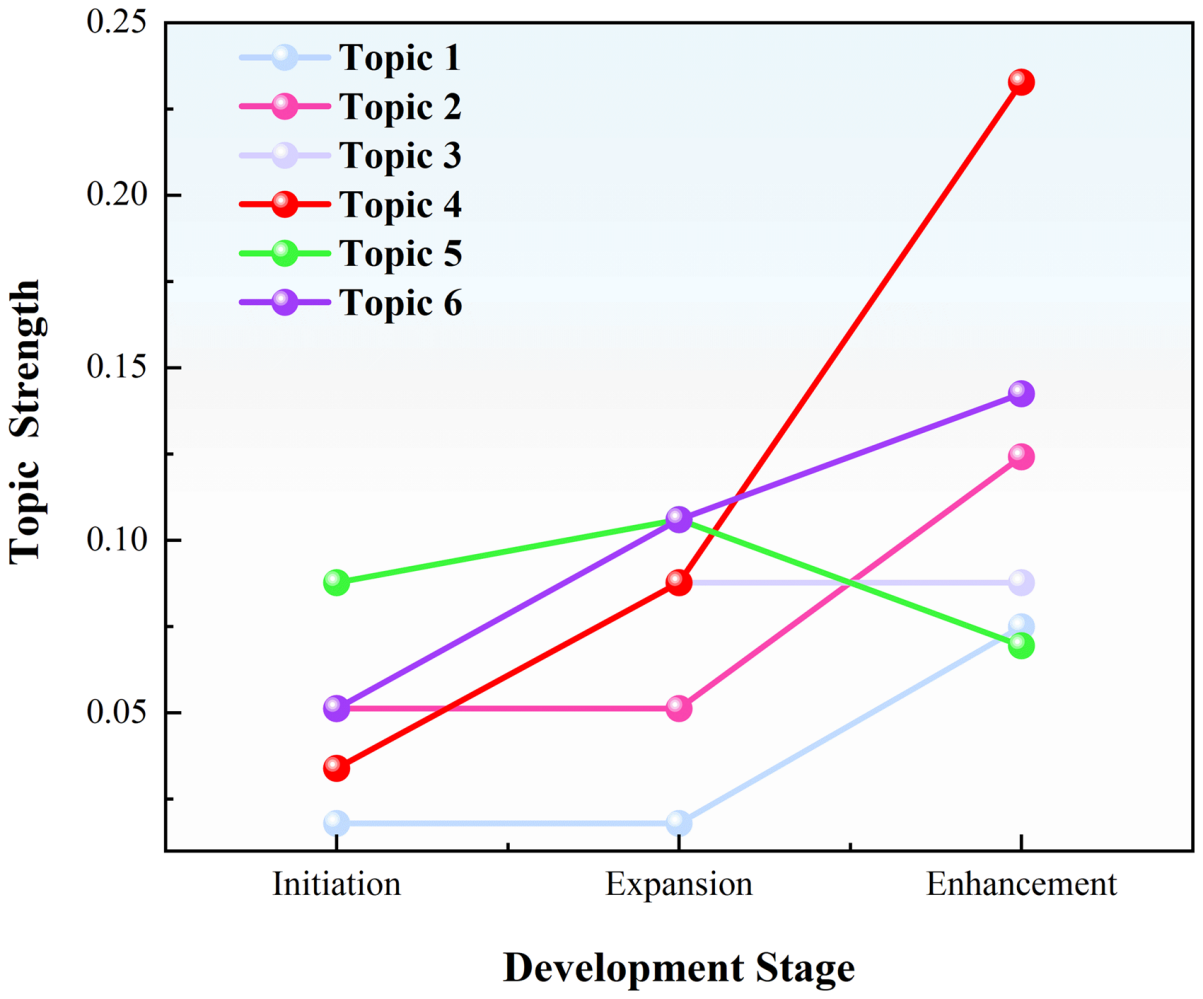
Temporal evolution of policy theme intensity.

To further explore the dynamic evolution of policy focus, this study introduces the “theme intensity” indicator to quantify the proportion of occurrence for six themes across different periods, and draws trend curves for the three distinct stages of the NFP from 1995 to 2025 (see Figure 5). This figure reveals the stage-specific fluctuations and temporal distribution characteristics of different themes during the policy evolution process, providing visual evidence for understanding the development path and focus shifts of the NFP.

#### 3.2.1 Theme 1: Policy leadership and infrastructure development

Keywords such as “sports industry,” “reform,” “training,” “athletes,” “government,” “system,” and “technology” reflect the importance the state places on top-level design and infrastructure development for NFP. The government leads the construction of the NFP system through institutional and policy frameworks emphasizing macro-control and policy safeguards. For instance the Chinese government launched the “weight management year” in 2024 with various regions implementing corresponding measures reflecting the core role of top-level design in policy promotion. Although this theme is strategically significant in terms of semantics its intensity remains relatively low across all three stages with a slight increase during the “enhancement stage,” indicating that as the NFP evolves its dominance is gradually shared with other functional themes.

#### 3.2.2 Theme 2: Elite sports and industry upgrading

Keywords such as “events,” “Olympics,” “winter sports,” “innovation,” “strong nation,” “structure,” and “model” emphasize the dual goals of the policy: Developing elite sports and driving the structural transformation of the sports industry. The development of elite sports under the national system is a characteristic of China’s sports policy. Currently elite sports lead the development of mass sports creating an environment for physical activity and achieving coordinated development between elite and mass sports. The figure shows a significant increase in intensity for this theme during the “enhancement stage,” especially after the 2022 Beijing winter Olympics highlighting the substantial impact of major sporting events on policy hotspots and their amplifying effect.

#### 3.2.3 Theme 3: Public health service system

Keywords such as “sports facilities,” “medical,” “health,” “public services,” “sports-medical integration,” “indicators,” and “capacity” reflect the policy’s extension toward health governance under the “healthy China” strategy emphasizing equity and inclusiveness. As shown in the figure the intensity of this theme has remained stable since the “expansion stage” with no significant increase or decrease indicating that the policy focus has remained consistent but has not become a dominant area.

#### 3.2.4 Theme 4: Event economy and social atmosphere building

Keywords such as “events,” “media,” “volunteers,” “promotion,” “mass sports,” and “lifestyle” indicate that the policy encourages participation through events and fosters a fitness atmosphere for all. This theme has shown the most rapid growth in the time series peaking during the “enhancement stage,” demonstrating the effective integration of event mechanisms and social mobilization and becoming a highlight of recent NFP policies. According to the 2024 Chinese Marathon data published by the China athletics association 749 marathons were held nationwide in 2024 with 7.0486 million participants an increase of 1 million from 2023. For example, the 2024 Wuxi Marathon saw 388,000 participants directly generating an economic benefit of 280 million RMB and tripling the foot traffic at nearby tourist attractions. This reflects the synergistic value of events in promoting healthy behaviors and driving sports tourism. This theme is especially prominent before and after major event cycles reflecting the policy’s focus on “soft mobilization” and the mechanism for mass participation.

#### 3.2.5 Theme 5: Legal and regulatory management

Keywords such as “regulations,” “systems,” “supervision,” “responsibility,” “measures,” “contracts” and “periodic” reflect the institutional orientation of policy governance. In 2009 to promote the development of mass fitness activities protect citizens’ legal rights in fitness activities and improve public physical fitness the Chinese government issued the National Fitness Regulations the first administrative regulation on NFP which defined fitness as a statutory right of citizens and designated august 8th as National Fitness day. The figure shows that the intensity of this theme decreased in the “enhancement stage” following a steady increase in earlier stages. This indicates that while legal governance remains a policy direction its weight has been replaced by more mobilizing and service-oriented themes in the new stage.

#### 3.2.6 Theme 6: Community sports infrastructure and policy support

Keywords such as “families,” “communities,” “volunteers,” “fitness equipment” and “financial support” reflect the trend of policy focus shifting toward grassroots levels. The figure clearly shows that this theme has been continuously rising across the three stages with a significant increase during the “enhancement stage,” reflecting the policy’s continued focus on providing fitness services at the “last mile” and highlighting the current focus on advancing public fitness services.

The study found that the themes of China’s NFP exhibit distinct phase-specific evolutionary characteristics: event economy and community sports support have significantly increased in recent years, reflecting a gradual shift in policy focus toward social mobilization and grassroots services; Elite sports and industry upgrading have shown fluctuations and increased intensity during Olympic cycles, reflecting the policy-driven effects of major sporting events; The health service system has remained stable, indicating its foundational role; The policy leadership and legal governance theme has stabilized after the initial establishment of the system, showing that the policy system has gradually shifted from macro design to execution and optimization.

### 3.3 Policy tool analysis (Y dimension)

**Figure 6:**
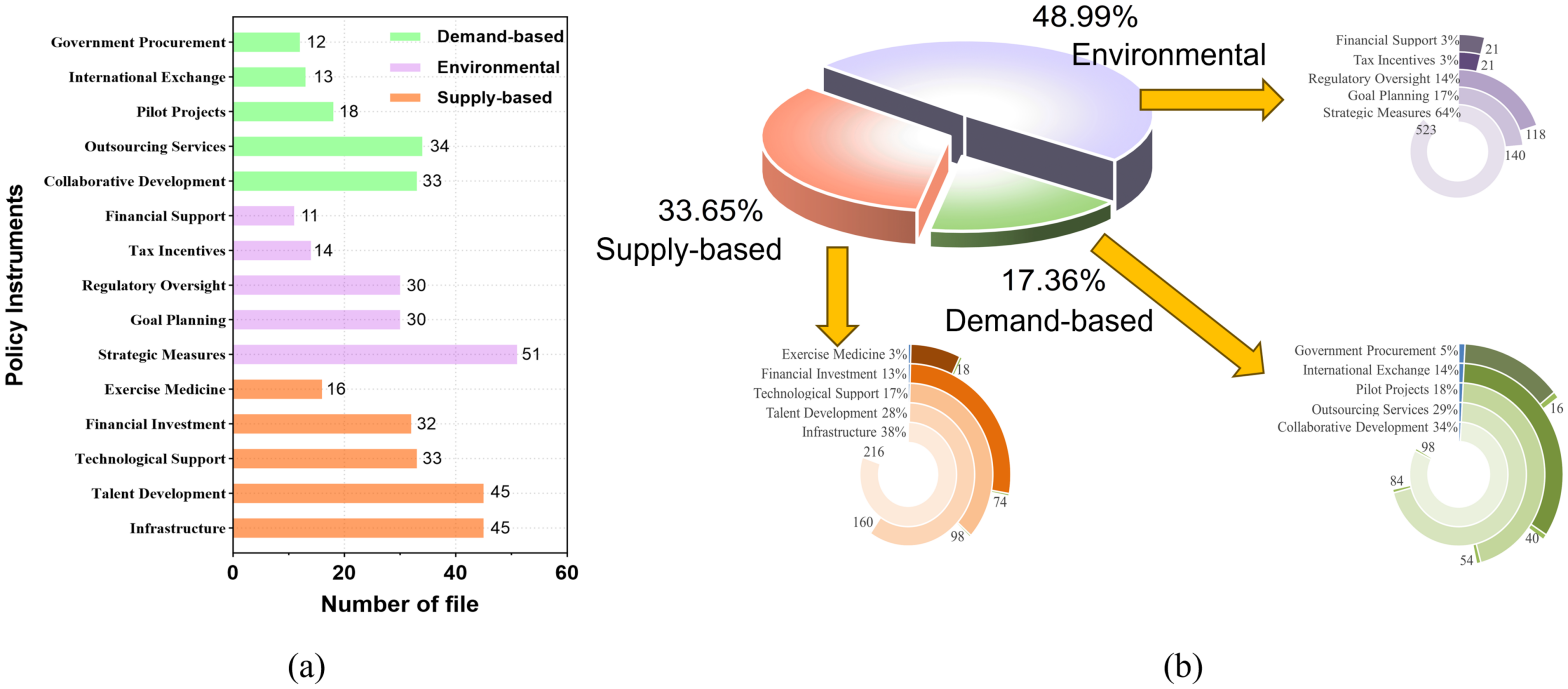
Distribution of policy tool types and frequency of use in the NFP. **(a)** Frequency statistics of policy tool usage. This figure presents the frequency of usage for 15 subcategories of the three main policy tool types in 52 policy documents. The results show that supply-side and environmental tools are used more frequently, especially “infrastructure,” “talent development,” and “strategic measures,” while demand-side tools are used less overall. **(b)** Proportion of policy tool structure his figure shows the overall proportions of supply-side, environmental, and demand-side policy tools, which are 33.65, 48.99, and 17.36%, respectively. The internal structure of the three tool types is further detailed in the rose chart, highlighting the current NFP policy tool combination’s bias toward environmental and supply-side tools, with relatively insufficient demand-side incentive mechanisms.

Supply-side, environmental, and demand-side policy tools play different roles in promoting, influencing, and driving the development of NFP. By categorizing and coding the content of 52 NFP texts, a total of 1,682 references for policy tool usage were identified. Of these, 52 policies used environmental policy tools, 50 policies used supply-side policy tools, and 46 policies used demand-side policy tools (see Figure 6a). This suggests that some policy texts have missing elements or incomplete structures in their use of policy tools. In terms of the proportion of tool types, environmental policy tools are the most commonly used, accounting for 48.99% (824), followed by supply-side tools at 33.65% (566), while demand-side tools account for only 17.36% (292; see Figure 6b). This indicates that the current NFP in China exhibits a dominant use of environmental policy tools, supplemented by some supply-side tools and fewer demand-side tools. This structure reveals a clear imbalance in the tool combination, particularly the lack of demand-side incentive mechanisms and the overemphasis on environmental tools. This may result in a policy implementation highly dependent on government leadership, with insufficient market and social involvement, thereby reducing the efficiency of policy implementation and the vitality of public participation. As previous studies have pointed out, the imbalance in the selection structure of policy tools in public policies often weakens the systemic coupling and sustained momentum of policy outcomes.

In supply-side policy tools, the usage proportions of infrastructure and talent development are 38.16 and 28.27%, respectively. This reflects the government’s focus on improving the public fitness service system through infrastructure development and the cultivation of fitness instructors. Over the past 30 years, China has made significant progress in constructing its NFP public service system. According to the 2024 National Sports Facility Statistical Survey published by the General Administration of Sport of China in March 2025, by December 31, 2024, there were 4.8417 million sports facilities in China, an increase of 4.226 million compared to when the NFP was launched in 1995 (70). The per capita sports facility area grew from 0.65 square meters in 1995 to 3.0 square meters, and the total number of national social sports instructors reached 3.71 million. However, due to the economic imbalance between eastern and western regions in China and the relatively low use of financial policy tools (13.07%), underfunded areas face insufficient financial support for NFP activities and uneven distribution of sports talent. This has led to regional disparities in the quality of public fitness services (35). The usage of Physical-medical integration is only 3.18%. The “Healthy China 2030” Planning Outline, issued by the State Council in 2016, elevated physical-medical integration to a national strategy. The limited use of this policy tool is related to barriers between China’s sports management departments and the healthcare system, as well as a lack of collaboration, which hinders the development of health promotion programs. This structural imbalance may lead to a concentration of resources in infrastructure construction and personnel allocation, while neglecting the establishment of long-term incentive mechanisms. As a result, the policy effect is more focused on “material supply” rather than “individual participation,” making it difficult to effectively stimulate grassroots enthusiasm and social collaboration.

There is an imbalance within environmental policy tools, with strategic measures accounting for 64% of the total, showing a clear bias. The advantage of strategic measures lies in their flexibility and operability, allowing for the implementation of NFP activities through specific action plans and measures. However, their drawback is that they focus on addressing current issues. Overreliance on these measures may lead to inconsistent policy execution and hinder the sustainable development of the NFP. The absence of enforcement and regulatory policies reduces the mandatory and binding force of the NFP, leading to insufficient policy implementation at the grassroots level. The use of financial support and tax incentive policy tools accounts for only 2.55%, and the lack of these tools hinders the participation of social organizations, businesses, and other social forces in the NFP. The insufficiency of financial and tax incentives limits the effective involvement of social capital, suppressing the diversification of the fitness industry and public goods supply mechanisms, and particularly hindering the formation of a “government-led—market-participation” collaborative governance model.

The use of demand-side policy tools is generally limited. Internally, collaborative development accounts for 35.56%, while service outsourcing and government procurement form a complementary combination, together accounting for 34%. International exchange has a relatively low share, only 2.38%. The high proportion of collaborative development reflects the government’s emphasis on building cross-departmental collaboration mechanisms. It promotes sustainable economic, social, and environmental development through regional integration, “sports-education integration,” “sports-medical integration,” and “sports-tourism integration.” International exchange helps enhance China’s international influence and promotes cultural communication and exchange. The limited use of this policy tool indicates that there is significant room for improvement in the openness of the NFP in terms of international cooperation. Service outsourcing and government procurement account for 29 and 5%, respectively. The lower use of government procurement indicates that the government prefers market-based procurement services to replace direct procurement of materials. For instance, Shanghai encourages social participation by outsourcing the operation of public sports venues, improving the quality and management of NFP services, and maximizing resources and social benefits. However, this may lead to imbalances in public sports services (with remote areas and low-income populations having limited access) and issues with the lack of service quality supervision. Overall, the lack of demand-side policy tools limits the development space and resource channels for NFP, making it difficult to generate the endogenous momentum for sustainable development of the NFP. Currently, the NFP policy tool system is relatively weak in terms of incentives and market mechanisms, leading to a greater reliance on government supply in policy implementation and a lack of grassroots vitality and feedback loops. This somewhat limits the institutional deepening and long-term operation of the NFP, and there is an urgent need to enhance the social mobilization effect and sustainability of the policy by expanding demand-side tools and optimizing resource allocation models.

### 3.4 Policy consistency analysis (Z dimension)

#### 3.4.1 Overall policy consistency analysis

Based on the previously described PMC index model calculation formula, the PMC indices, rankings, and evaluation levels of 12 NFP texts were determined (see Table 6). Among them, the highest-scoring policy text is P9 (7.61), while the lowest is P3 (4.12). Based on the scores, the policy texts are classified into two evaluation levels: policies with a “Good” rating include P4, P6, P7, P9, P10, and P12, while those with an “Average” rating include P1, P2, P3, P5, P8, and P11. Notably, no policy text received a “Poor” or “Excellent” rating. The average PMC index of the 12 policies is 6.06/10, with a “Good” rating, indicating that the overall design of China’s NFP is relatively scientific and reasonable.

**Table 6:** Inputs and outputs of 12 NFP policies.

	P1	P2	P3	P4	P5	P6	P7	P8	P9	P10	P11	P12	Mean
X1	1	0.5	0.33	1	0.5	0.83	0.5	0.5	0.83	0.67	0.67	0.67	0.67
X2	0.33	0.67	1	1	1	1	1	0.67	0.67	1	0.67	1	0.83
X3	0.8	0.8	0.4	1	0.8	0.8	0.8	1	1	0.8	1	1	0.84
X4	0.67	0.43	0.57	0.71	0.43	0.86	0.71	0.71	0.86	0.86	0.86	1	0.72
X5	0.4	0.4	0.4	0.6	0.6	0.8	0.8	0.4	1	0.6	0.4	1	0.62
X6	0.88	0.63	0.14	0.71	1	0.83	1	0.86	1	0.5	0.63	0.75	0.74
X7	0.2	0.4	0.6	0.6	0.4	0.8	0.6	0.6	1	0.8	0.6	0.8	0.62
X8	0.25	0.25	0.25	0.25	0.25	0.25	0.25	0.25	0.25	0.25	0.25	0.25	0.25
X9	0.71	0.86	0.43	0.86	0.43	0.86	0.71	0.86	1	0.86	0.57	1	0.76
PMC	5.24	4.94	4.12	6.73	5.41	7.03	6.37	5.85	7.61	6.34	5.65	7.47	6.06
Rank	10	11	12	4	9	3	5	7	1	6	8	2	
Level	average	average	average	good	average	good	good	average	good	good	average	good	good

#### 3.4.2 Longitudinal analysis of each period

**Figure 7:**
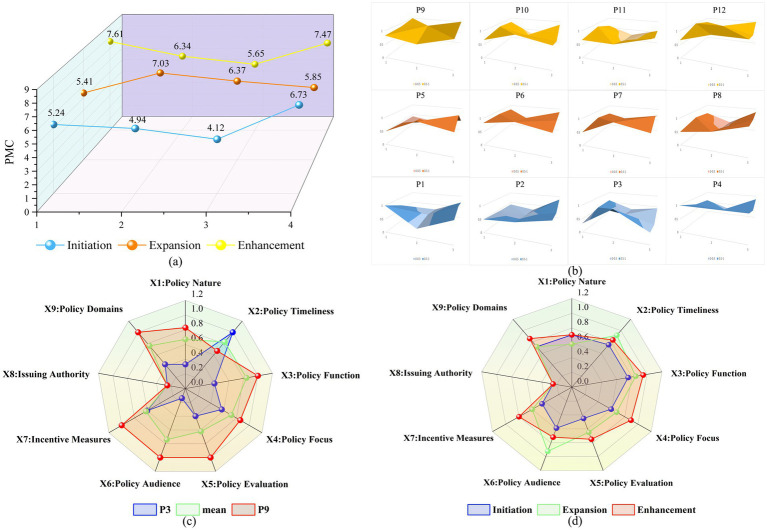
PMC index evaluation results of NFP policies. **(a)** PMC average trend of policies across different development stages. This figure displays the trend of PMC averages for different representative policies across three stages: “Initiation Stage,” “Expansion Stage,” and “Improvement Stage.” It reflects the gradual improvement in policy design quality over time, with a notable increase during the “Improvement Stage.” **(b)** PMC surface plot of 12 representative policies. This figure shows the structural characteristics of 12 representative policies based on the nine primary PMC indicators. The shape differences highlight the varying emphases of each policy in terms of function, incentives, timeliness, and other aspects. **(c)** PMC radar comparison of representative policies P3 and P9. This figure compares the PMC scores of the lower-scoring policy (P3) and the higher-scoring policy (P9) with the average value, emphasizing the strengths of high-scoring policies in terms of incentives, supervision, policy functions, and other aspects. **(d)** Average PMC radar of policies in three stages. This figure displays the average performance of representative policies from each stage, revealing improvements in most indicators during the “Improvement Stage,” particularly in “Policy Evaluation,” “Audience Coverage,” and “Incentive Mechanisms”.

Although the overall effectiveness of the 12 policy texts is rated as “Good,” Figures 7a,b show significant fluctuations in the PMC score trends across different periods, reflecting score variations in policies at different stages. For example, during the improvement phase, although P11 and P12 were both issued by the State Council and only 8 months apart, P12 scored 1.82 points higher than P11, showing that even policies issued by the same institution may result in significant differences due to slight variations in policy design and implementation. Figure 7c visually displays the score differences across primary indicators (such as policy nature, function, and incentive mechanisms) between the highest-scoring policy (P9) and the lowest-scoring policy (P3), reflecting significant disparities in design quality between policies. The average PMC scores for the three periods were 5.26, 6.17, and 6.77, respectively. Combined with Figures 7a,d, it can be seen that the policy consistency level gradually improved across the three stages. Despite differences between individual policies, the overall trend shows that, with the accumulation of practical experience and deepening policy theoretical research, China’s NFP has gradually improved, with a steady enhancement in design quality. However, some indicators still require significant optimization.

#### 3.4.3 Horizontal comparative analysis of variables

Based on the average scores across different indicators (Figure 7c), X1 Policy Nature (0.67), X5 Policy Evaluation (0.62), X7 Incentive Measures (0.62), and X8 Issuing Authority (0.25) have lower scores, which lowers the overall consistency score. By comparing the primary indicator scores of each policy with the mean score, the strengths and weaknesses across different aspects are analyzed, and the gaps in the policy texts are identified. In X1 Policy Nature, five policies scored below the average. The low score in X1 is mainly due to the lack of predictive content in 7 policies and diagnostic content in 6 policies. The value of predictive policies lies in helping decision-makers focus on core issues, ensuring scientific decision-making, avoiding blind decisions, and enhancing foresight while reducing risks through long-term planning. The absence of this content may lead to policies being unable to flexibly respond to future changing external environments and challenges. The lack of diagnostic content can lead to inefficient allocation of public fitness resources and reduced policy precision, ultimately affecting policy implementation. These deficiencies further reveal that the policy lacks the ability to address future uncertainties and optimize resource allocation, which may exacerbate inequalities between regions and groups, particularly in resource-constrained situations.

The low score in X5 is primarily related to the lack of content on sustainable development (X5:5). Only three policy texts from the “Improvement Stage” (P9, P10, P12) mention the need for NFP activities to focus on coordinating economic, social, and environmental development, following a sustainable development path. The new development concept proposed by President Xi Jinping in 2015, emphasizing innovation, coordination, green development, openness, and sharing, particularly highlights that green development is essential for sustainability and actively addresses the issue of harmonious coexistence between humans and nature. This also indicates that NFP activities, guided by the new development concept, have increasingly focused on integrating with sustainable development goals after entering the Improvement Stage. However, policies that lack sustainability considerations struggle to maintain long-term effectiveness, particularly in areas such as resource allocation, environmental protection, and social equity, which can limit the effectiveness and impact of policy execution.

In X7 Incentive Measures, eight policies scored below the average. Analysis shows that only four out of the 12 policies involve tax incentives. The lack of this content limits the input of social resources and hinders the encouragement of social participation in the development of NFP. Policies that lack incentive measures struggle to mobilize the private sector and social organizations, often relying on one-sided government input, which may lead to an imbalance in resource allocation.

The score for X8 Issuing Authority is only 0.25, mainly due to the fact that all 12 policies were issued by a single department, lacking inter-departmental collaboration and coordination during the policy formulation process. Single department leadership results in policy design limitations, and the lack of a cross-departmental coordination mechanism may cause policy implementation to lack integration. This could lead to redundancy or gaps in some areas, making it difficult to integrate resources from health, education, sports, and other sectors, thus limiting policy effectiveness. Additionally, X4 Policy Focus, as the core indicator for evaluating policy consistency, covers a limited number of policies related to sports-medical integration and international exchanges. Sports-medical integration, an important path for achieving national health, is covered in only three policies, indicating barriers to inter-departmental cooperation, poor information sharing, and unclear responsibilities, which hinder resource integration and collaborative innovation across sectors. This lack of cooperation and coordination in the structure may lead to fragmented policy effects, affecting the long-term sustainability and execution of NFP.

Analysis of the data reveals that China’s NFP performs well in X2 Policy Timeliness, balancing the coordination of short-term and long-term development. The high score for X6 Policy Audience reflects the Chinese government’s emphasis on the public fitness service system, particularly in improving the physical health levels of all citizens. However, there is still a lack of detailed discussion on how to ensure that NFP services benefit every citizen, particularly regarding how to ensure equitable coverage and regional balance. There is a lack of specific implementation details and monitoring mechanisms, leading to uneven implementation effects across regions and populations.

## 4 Conclusion and discussion

### 4.1 Research findings

This study combines qualitative and quantitative methods to analyze China’s NFP policy texts from three dimensions: policy themes, policy tools, and policy consistency. The study found that although China’s NFP has a certain degree of rational design, there are still issues in policy tool configuration, balance of tools, and policy consistency, which affect the implementation effectiveness of the policies.

(1) The Relationship Between Policy Themes and Tool Selection

China’s NFP has constructed a policy framework centered around infrastructure development and mass sports activities in its overall design. The policy emphasizes infrastructure development and service guarantees to promote widespread participation in NFP. However, policy tool analysis shows that although the policy focuses on infrastructure development, the use of demand-side tools is insufficient, particularly in cross-departmental cooperation and market incentives. Theme analysis indicates that Theme 3, “Health Service System” requires more cross-departmental tools (such as sports-medical integration), but the use of such tools is still limited, further restricting the comprehensiveness and effectiveness of the policy. The combination of tool and theme analysis indicates that the imbalance in tool configuration may limit social mobilization and participation, affecting the long-term implementation of the policy.

(2) The Impact of Policy Tool Imbalance

Policy tool analysis shows that environmental tools (such as strategic measures and planning goals) dominate the NFP, accounting for 48.99%, while the use of demand-side tools is much lower, at 17.36%. Although environmental tools promote the implementation of NFP through strategic planning and policy frameworks, overreliance on these tools may lead to a lack of market incentives and effective social participation, restricting the long-term development of the policy. Especially, government procurement usage is only 0.95%, indicating that the policy relies on a single approach to incentivize the private sector and social organizations, failing to effectively mobilize market dynamics. Additionally, while supply-side tools play a positive role in infrastructure development, the lack of sufficient demand-side tools (such as tax incentives and market incentives) limits social mobilization and market participation. This imbalance in tools leads to a “government-led, market-cold, public-waiting” dilemma in policy implementation.

(3) Analysis of Policy Consistency and Effectiveness

China’s NFP is generally scientifically and reasonably designed, but there are differences in policy consistency across different policy texts. In terms of policy nature, most policies are advisory and lack predictive and diagnostic content. Predictive policies help decision-makers respond to future changes and ensure policy flexibility. The lack of such content makes it difficult for policies to effectively address future changes and challenges. The absence of diagnostic content may lead to inaccurate resource allocation and affect the efficiency of policy execution. In terms of incentives, the policy focuses on talent incentives but has fewer tax incentives, which limits social participation and suppresses the full mobilization of social capital and market mechanisms. In terms of policy issuance, the policies are mainly issued by a single department, lacking cross-departmental collaboration. This may lead to a lack of coordination and consistency during implementation, affecting the policy’s effectiveness. By combining policy theme analysis, tool analysis, and policy consistency evaluation, we found that while China’s NFP has certain advantages in design, there are still significant issues such as policy tool imbalance and insufficient consistency. Over-reliance on supply-side tools and the absence of demand-side tools have made it difficult to effectively mobilize social participation, limiting the long-term sustainability of the policy. At the same time, insufficient cross-departmental collaboration and a lack of policy incentives may affect the implementation effectiveness and realization of NFP goals.

### 4.2 Research recommendations

Based on the above findings, this study proposes the following policy improvement recommendations: (1) Enhance cross-departmental cooperation and communication. In the formulation and implementation of the NFP, communication and cooperation between departments such as sports, healthcare, and education should be further strengthened. For example, it is recommended to jointly establish a comprehensive public health talent cultivation system and promote active participation from all stakeholders by establishing cross-departmental cooperation mechanisms. By building a data-sharing platform and utilizing technologies such as big data and cloud computing, the scientific validity, rationality, and comprehensiveness of policy formulation can be improved. Additionally, the government could hold regular cross-departmental meetings or set up joint working groups to ensure coordination and consistency in the implementation process. For example, Finland’s national fitness policy system has a clear policy collaboration mechanism, emphasizing the complementarity and synergy of various government departments in policy implementation. Multiple departments, including health, education, and transportation, work closely together in areas such as public health and sports facility construction, jointly promoting the construction of sports facilities and the popularization of national fitness activities. (2) Focus on the inclusivity and regional balanced development of public fitness services. NFP should pay more attention to the inclusivity of the public fitness service system and promote regional balanced development. It is recommended to increase special funding investments and talent incentives to promote the participation rights of different regions, classes, and groups (especially people with disabilities, farmers, the older adult, low-income populations, and youth) in sports activities. Strengthen the coverage of supply-side policy tools (such as infrastructure development) in weaker regions to ensure the fairness of public fitness services across urban and rural areas and regions (71, 72). (3) Enhance demand-side incentive measures and promote social participation. Demand-side incentive measures such as tax incentives should be added to the policy tools. For instance, government subsidies and venue rent reductions can be used to mobilize market forces and attract social organizations and businesses to participate in the NFP. It is recommended to offer tax breaks for companies providing employee fitness programs or provide fitness subsidies (e.g., gym membership vouchers) to individuals, effectively stimulating market and social participation. These measures can strengthen the social mobilization effect of the NFP and create a synergy between the government and society. For example, private fitness clubs and fitness equipment manufacturers in the United States, guided by favorable policies such as tax incentives and subsidies, have provided a large number of socialized sports services. This has not only increased public participation but also promoted the growth of related industries. (4) Increase the number of demand-side policy tools. There is a need to increase the number of demand-side policy tools, such as service outsourcing, government procurement, and international exchanges, to further implement the NFP. Service outsourcing and government procurement can attract social forces and businesses, offering broader support and guarantees for the NFP. At the same time, international exchange can help learn from successful global experiences and enhance policy implementation effectiveness. (5) Improve the supervision and evaluation system for local government policy execution. The government should establish an evaluation index system for the effectiveness of NFP policy execution through research institutes and third-party organizations. It is recommended to conduct regular fitness surveys, set specific performance indicators for local governments, and establish a feedback mechanism to adjust policies in a timely manner based on the evaluation results. The supervision mechanism for policy execution can ensure continuous optimization and adjustment of policies during implementation, ensuring the sustainability of the policy. For example, the UK’s Health Lifestyle Promotion Program continually improves the details of policy implementation through regular assessments and data collection, ensuring that policy measures align with actual needs. (6) Strengthen international cooperation and promote global collaborative development. In the promotion of health programs, international cooperation should be strengthened, and an international cooperation platform should be established to promote resource and experience sharing and mutual assistance. Joint research, policy exchanges, and international project cooperation can be utilized to learn from successful global experiences. At the same time, international organizations and governments should be encouraged to develop standardized policy frameworks to lay the foundation for global collaborative development.

### 4.3 Research limitations

This study is based on 52 NFP policy documents from China, and constructs a three-dimensional analysis framework of “policy themes—policy tools—policy design consistency” to systematically reveal the structural logic and evolutionary trends of the NFP. However, the study has the following limitations: Firstly, although the PMC index model reduces subjectivity by assigning equal weights to all indicators to ensure fairness in the evaluation process, it should be noted that not all policy elements have the same importance regarding health outcomes. Therefore, the assumption in the PMC model (that all indicators are equally important) may introduce bias into the results in some cases. Secondly, this study focuses only on national-level policy documents issued by the CPC Central Committee, the State Council, and relevant ministries, excluding policy texts from provincial, municipal, or district levels. Therefore, it may not fully capture the differentiated paths, adaptive adjustments, or grassroots innovations in policy implementation by local governments. Thirdly, the analysis in this study is based solely on policy texts themselves, focusing on the rationality of policy structure design and tool configuration. It does not combine empirical evaluation based on actual performance in policy implementation, making it difficult to fully reflect the real-world effects of policy deployment. Future research can expand the data sources and analytical dimensions of this study, incorporating local policy texts, execution data, and audience feedback. This will allow for a deeper exploration of the practical operation mechanisms and implementation effectiveness of the NFP, thus providing a more comprehensive presentation of the policy lifecycle.

## Data Availability

The datasets presented in this study can be found in online repositories. The names of the repository/repositories and accession number(s) can be found in the article/supplementary material. This data can be found here: https://www.gov.cn/.
